# FCER1G positively relates to macrophage infiltration in clear cell renal cell carcinoma and contributes to unfavorable prognosis by regulating tumor immunity

**DOI:** 10.1186/s12885-022-09251-7

**Published:** 2022-02-04

**Authors:** Keqin Dong, Wenjin Chen, Xiuwu Pan, Hongru Wang, Ye Sun, Cheng Qian, Weijie Chen, Chao Wang, Fu Yang, Xingang Cui

**Affiliations:** 1 Department of Urinary Surgery, Third Affiliated Hospital of Naval Medical University (Eastern Hepatobiliary Surgery Hospital), 700 North Moyu Road, 201805 Shanghai, China; 2 Post-graduate Training Base in Shanghai Gongli Hospital, Post-graduate College, Ningxia Medical University, Yinchuan, China; 3 Department of Urinary Surgery, Gongli Hospital, Second Military Medical University (Naval Medical University), 219 Miaopu Road, 200135 Shanghai, China; 4 The Department of Medical Genetics, Naval Medical University, 800 Xiangyin Road, 200433 Shanghai, China

**Keywords:** Clear cell renal cell carcinoma, Bioinformatic analysis, Tumor-associated macrophage, FCER1G, Prognosis, Tumor microenvironment

## Abstract

**Background:**

Tumor-associated macrophages (TAMs) are closely related to unfavorable prognosis of patients with clear cell renal cell carcinoma (ccRCC). However, the important molecules in the interaction between ccRCC and TAMs are unclear.

**Methods:**

TCGA-KIRC gene expression data of tumor tissues and normal tissues adjacent to tumor were compared to identify differentially expressed genes in ccRCC. TAMs related genes were discovered by analyzing the correlation between these differentially expressed genes and common macrophage biomarkers. Gene set enrichment analysis was performed to predict functions of TAMs related gene. The findings were further validated using RNA sequencing data obtained from the CheckMate 025 study and immunohistochemical analysis of samples from 350 patients with ccRCC. Kaplan–Meier survival curve, Cox regression analysis and Harrell’s concordance index analysis were used to determine the prognostic significance.

**Results:**

In this study, we applied bioinformatic analysis to explore TAMs related differentially expressed genes in ccRCC and identified 5 genes strongly correlated with all selected macrophage biomarkers: *STAC3*, *LGALS9*, *TREM2*, *FCER1G*, and *PILRA*. Among them, *FCER1G* was abundantly expressed in tumor tissues and showed prognostic importance in patients with ccRCC who received treatment with Nivolumab; however, it did not exhibit prognostic value in those treated with Everolimus. We also discovered that high expression levels of *FCER1G* are related to T cell suppression. Moreover, combination of *FCER1G* and macrophage biomarker *CD68* can improve the prognostic stratification of patients with ccRCC from TCGA-KIRC. Based on the immunohistochemical analysis of samples from patients with ccRCC, we further validated that FCER1G and CD68 are both highly expressed in tumor tissue and correlate with each other. Higher expression of CD68 or FCER1G in ccRCC tissue indicates shorter overall survival and progression-free survival; patients with high expression of both CD68 and FCER1G have the worst outcome. Combining CD68 and FCER1G facilitates the screening of patients with a worse prognosis from the same TNM stage group.

**Conclusions:**

High expression of *FCER1G* in ccRCC is closely related to TAMs infiltration and suppression of T cell activation and proliferation. Combining the expression levels of FCER1G and macrophage biomarker CD68 may be a promising postoperative prognostic index for patients with ccRCC.

**Supplementary Information:**

The online version contains supplementary material available at 10.1186/s12885-022-09251-7.

## Background

The incidence of renal cell carcinoma (RCC) has been increasing as a result of the routine application of abdominal imaging. Clear cell renal cell carcinoma (ccRCC) is the most common pathological subtype of RCC, accounting for approximately 75% of all RCC cases and most cancer-related deaths [1]. Surgical resection is the standard treatment for localized ccRCC. Nevertheless, 25–40% of patients develop metastatic lesions even after radical treatment [2]. Following disease progression to an advanced state, the patients depend on tyrosine kinase inhibitors (TKIs) as first-line intervention; however, resistance to TKIs inevitably develops after standard treatment [3]. Therefore, an enhanced understanding of key regulatory molecules in ccRCC is urgently needed to more accurately estimate the long-term outcome of postoperative patients and develop more durable treatment options.

In recent years, the tumor immune microenvironment has attracted considerable research attention. Infiltration of immune-inhibitory cells is a hallmark of ccRCC that alters therapeutic effectiveness [4]. Clinical trials of immune checkpoint inhibitor (ICI) versus TKI treatment for ccRCC, such as CheckMate 214 [5], CheckMate 9ER [6], Keynote-426 [7], and IMmotion 151 [8] have shown pronounced clinical benefits. A recent update of the 4-year follow-up results of the CheckMate 214 study also showed durable efficacy [9], prompting researchers to reconsider the possibility of targeting tumor immunity as a long-term effective treatment for advanced ccRCC. However, a considerable proportion of patients showed limited reaction to ICI, while the underlying mechanism remains poorly understood [10].

Tumor-associated macrophages (TAMs) are key immune cell populations in ccRCC with great diversity in phenotype and functions [11]. They act through a variety of mechanisms to compromise the effects of treatment with TKIs and ICIs, including the promotion of angiogenesis [12], acceleration of tumor growth [13], and suppression of antitumor immunity [14]. We have previously reported that several molecules, such as SRY-box transcription factor 17 (SOX17) [15] and gankyrin [16], can regulate the function of TAMs to facilitate the progression of ccRCC. Hence, targeting TAMs is a potential option for developing treatments against ccRCC and obtaining a synergic effect from the utilization of TKIs and ICIs [17]. To determine the mechanism through which TAMs interact with ccRCC, we investigated whether other key molecules govern the biological function of TAMs in this disease. For this purpose, we used The Cancer Genome Atlas-kidney renal clear cell carcinoma (TCGA-KIRC) dataset to predict macrophage-related molecules and validated the findings in a large cohort of patients from the clinical setting.

## Methods

### Bioinformatics analysis

The RNA sequencing (RNA-seq) count data of 538 tumors and 72 normal tissues adjacent to tumor (NATs) from TCGA-KIRC were downloaded from the online database using the R package ‘TCGAbiolinks’ [18]. Array Intensity correlation analysis was performed using the ‘TCGAanalyze_Preprocessing’ function to remove outliers (cut-off correlation score: 0.6). The ‘TCGAtumor_purity’ function was used to exclude unqualified tumor samples (tumor purity level <60%). The count data of TCGA-KIRC were further normalized using the GC-content method [19]. To obtain a more rigorous outcome, genes with expression levels <25% of the average expression were excluded. Analysis of differentially expressed genes (DEGs) was performed using the R package ‘Limma’; the expression data were transformed as log (exp+1), while |log fold change| > 0.1 and adjusted *P*-value <1e-10 denoted significant differences in expression. Normalized bulk RNA-seq and clinical data of patients with ccRCC treated with either programmed cell death 1 (PD-1) blockade or mechanistic target of rapamycin kinase (MTOR) inhibition were obtained from the CheckMate 025 study [20]. To perform functional enrichment analysis, DEGs were loaded into clusterProfiler [21] for Gene Ontology and Kyoto Encyclopedia of Genes and Genomes enrichment analyses. Pathways with an adjusted *P*-value <0.05 were considered significantly enriched. Gene set enrichment analysis (GSEA) was conducted to detect significantly enriched gene sets. Only gene sets with false discovery rate and nominal *P*-values < 0.05 were considered significantly enriched.

### Patients and specimens

RCC tissue microarrays (TMAs) of 407 patients from Shanghai Changhai Hospital were included in this study; they involved two series. The first series (TMA-30) consisted of 29 patients with ccRCC (from our previous research) [15, 16], who underwent surgery from 2010 to 2015. Follow-up of all patients in TMA-30 via telephone interviews confirmed either disease progression (recurrence/metastasis) or cancer-related death. The TMA-30 microarray included 10 pairs of tumors and NATs together with 19 cases of unpaired tumor tissue; two replicates were performed for each included sample. For another series (TMA-2020 NO.1-8) 378 patients with RCC who underwent surgery from 2016 to 2018 were indifferently included in eight TMAs. Of all included patients in TMA-2020 NO.1-8, 321 had ccRCC according to pathological examination. TMA-2020 NO.1-8 consists of 270 pairs of ccRCC tumors and NATs together with 50 cases of unpaired tumor tissue. We were able to contact 266 of the 321 patients with ccRCC for follow-up through telephone interviews. The clinical data (e.g., age, sex, TNM stage, and Fuhrman grade) of both series are summarized in Table 1. (Additional table files show this in more detail (see Additional file 1 and 2)). The primary outcomes were overall survival (OS) and progression-free survival (PFS). OS was defined as the duration of follow-up from surgery to the date of cancer-related death. PFS was defined as the duration of follow-up from surgery to the date of clinically diagnosed metastasis or recurrence of ccRCC. Samples for the IHC positive control were kindly donated by the Pathology Department of Shanghai Changhai Hospital. All experiments were approved by the Scientific Research Review and Investigation Committee of the Third Affiliated Hospital of the Second Military Medical University (ID:EHBHKY2020-K-026, approval date: August 17^th^,2020 ), and written informed consent was provided by all patients.

**Table 1:** Characteristics of patients with clear cell renal cell carcinoma

Characteristics	Cohort description		Total (*n* = 350)
	TMA-30 (*n* = 29)	TMA-2020 NO.1-8 (*n* = 321)	
**Age, years**			
Mean±SD	60.3 ± 10.9	57.0 ± 11.9	57.2 ± 11.9
Median	63	55	56
Range	39–80	23–86	23–86
**Sex, n (%)**			
Male	24 (82.8)	224 (69.8)	248 (70.9)
Female	5 (17.2)	97 (30.2)	102 (29.1)
**Fuhrman grade, n (%)**			
1–2	18 (62.1)	261 (81.3)	279 (79.7)
3–4	11 (37.9)	60 (18.7)	71 (20.3)
**TNM stage, n (%)**			
I–II	23 (79.3)	275 (85.7)	298 (85.1)
III–IV	6 (20.7)	45 (14.0)	51 (14.6)
NA	0	1 (0.3)	1 (0.3)
**T category, n (%)**			
T1a–T1b	19 (65.5)	252 (50.5)	271 (77.4)
T2a–T4	10 (34.5)	68 (49.2)	78 (22.3)
NA	0	1 (0.3)	1 (0.3)
**Overall survival, n (%)**			
−	2 (6.9)	249 (77.6)	251 (71.7)
+	27 (93.1)	17 (5.3)	44 (12.6)
NA	0	55 (17.1)	55 (15.7)
**Progression-free survival, n (%)**			
−	5 (17.2)	236 (73.5)	241 (68.9)
+	24 (82.8)	30 (9.3)	54 (15.4)
NA	0	55 (17.1)	55 (15.7)

*NA* not applicable; *SD* standard deviation; *TMA* tissue microarray

### IHC

IHC assay was conducted as previously described [15]. Briefly, paraffin-embedded sections of ccRCC TMA were preprocessed with the antigen retrieval procedure. For the antibody against CD68, citric acid was used at a pH of 6.0. For the antibody against FCER1G, ethylene diamine tetraacetic acid was used at a pH of 9.0. Goat serum (10%) was used to block nonspecific binding. The slides were subsequently incubated overnight with antibodies against CD68 (#76437s, rabbit anti-human monoclonal, 1:400; Cell Signaling Technology) and FCER1G (ab151986, rabbit anti-human polyclonal, 1:400; Abcam) at 4°C in an incubator. After incubation with horseradish peroxidase-conjugated secondary antibodies (ab205718, goat anti-rabbit IgG polyclonal, 1:2000; Abcam) for 1 hour at room temperature the following day, slides were developed using 3,3'-diaminobenzidine staining for 2 min, and the cell nucleus was stained with hematoxylin. Human tonsil and spleen tissues were used as positive controls for CD68 and FCER1G, respectively (Data not shown). The ccRCC slides incubated only with secondary antibodies were used as negative control (Data not shown). All slides were scanned with Hamamatsu Nanozoomer S60 (Hamamatsu City, Japan), and evaluated on the same displayer using the NDP.view software (Version 2.9.20). Given that both CD68 and FCER1G are membrane surface markers that label single cells within tumors, the staining intensity was defined as the percentage of area covered by positive cells (0–100) multiplied by the density of positive cells in given areas (0–3). Using this standard, an IHC score of 0–300 was generated for statistical evaluation. All slides were independently evaluated by two experienced pathologists, who calculated the mean IHC score. The representative images were captured using Olympus microscope (model BX51) and processed using Adobe Photoshop and Illustrator (Version CC 2017).

### Statistical analysis

The two-tailed Student’s *t*-test or Wilcoxon test was conducted for continuous variables. The chi-squared test or Fisher’s exact test was conducted for categorical variables. The Kaplan–Meier method was used to draw survival curves using the “survival” package and “survminer” in R software version 3.6.3. Variables with a P-value <0.05 on the univariate analysis were included in the multivariate Cox regression analysis using the “survival” package of R 3.6.3. Receiver operating characteristics (ROC) analysis was performed to obtain the cut-off value and the area under the ROC curve (AUC) using the GraphPad Prism 7.00 software (GraphPad Software, Inc., San Diego, CA, USA). The prognostic accuracy of the strata and other clinical prognostic factors was calculated through Harrell’s concordance index analysis using the “survcomp” package in R software version 3.6.3. Nomogram analysis was conducted using the “foreign” (version 0.8-78) and “rms” (version 6.0.1) packages to establish the risk prediction model. All statistical analyses were performed using the R software (version 3.6.3) and GraphPad Prism (version 7.00).

## Results

### Identification of macrophage-related DEGs in ccRCC using TCGA-KIRC database

**Fig. 1:**
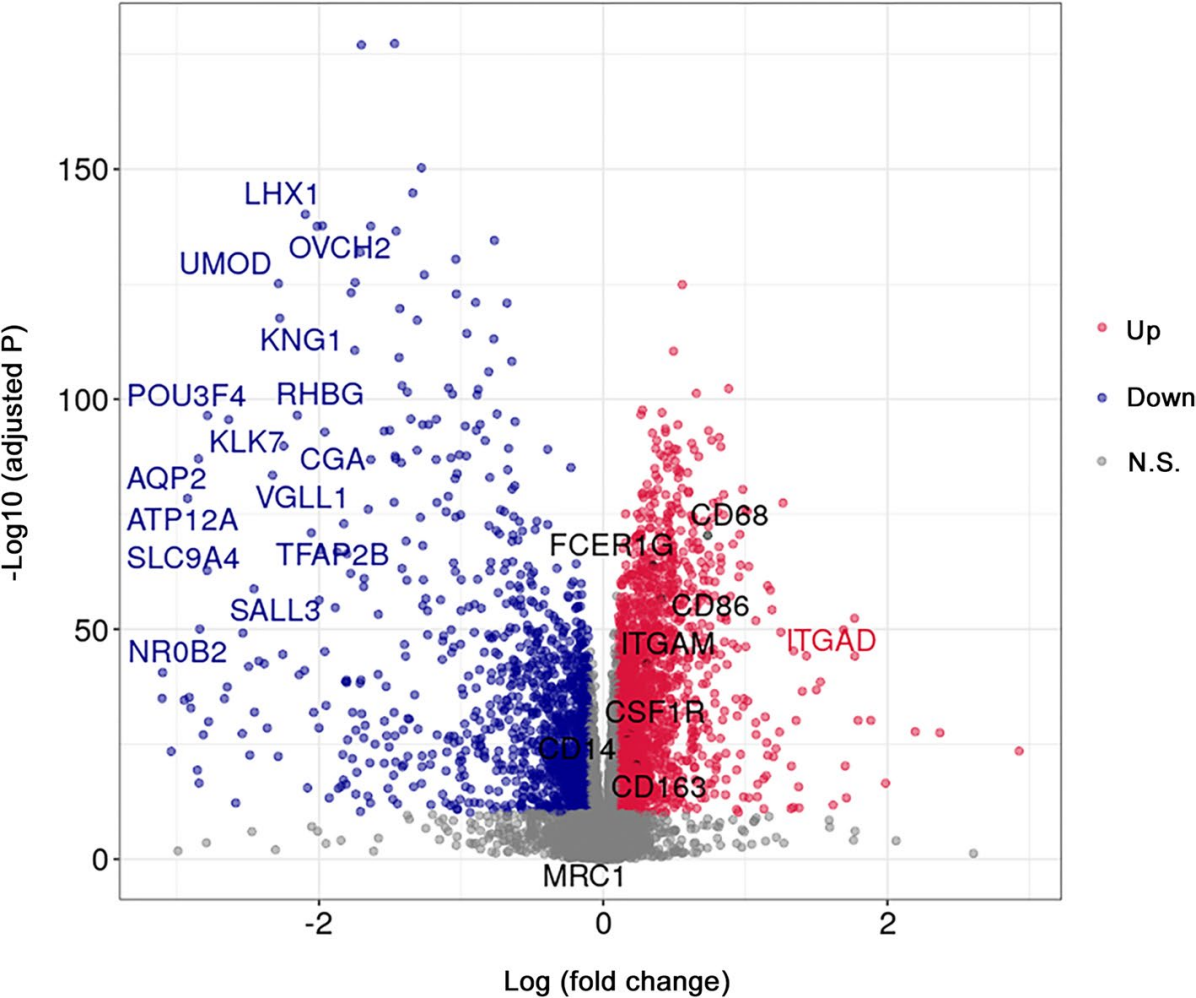
The differentially expressed genes (DEGs) in TCGA-KIRC database. Volcano plot of DEGs between tumor and NATs identified in ccRCC. The red and blue points in the plot represent DEGs with statistical significance (|logFC| >0.1, adjusted *P* < 1e-10) N.S.: No significance. ccRCC, clear cell renal cell carcinoma; logFC, log fold change; NATs, normal tissues adjacent to tumor; TCGA-KIRC, The Cancer Genome Atlas-kidney renal clear cell carcinoma;

**Fig. 2:**
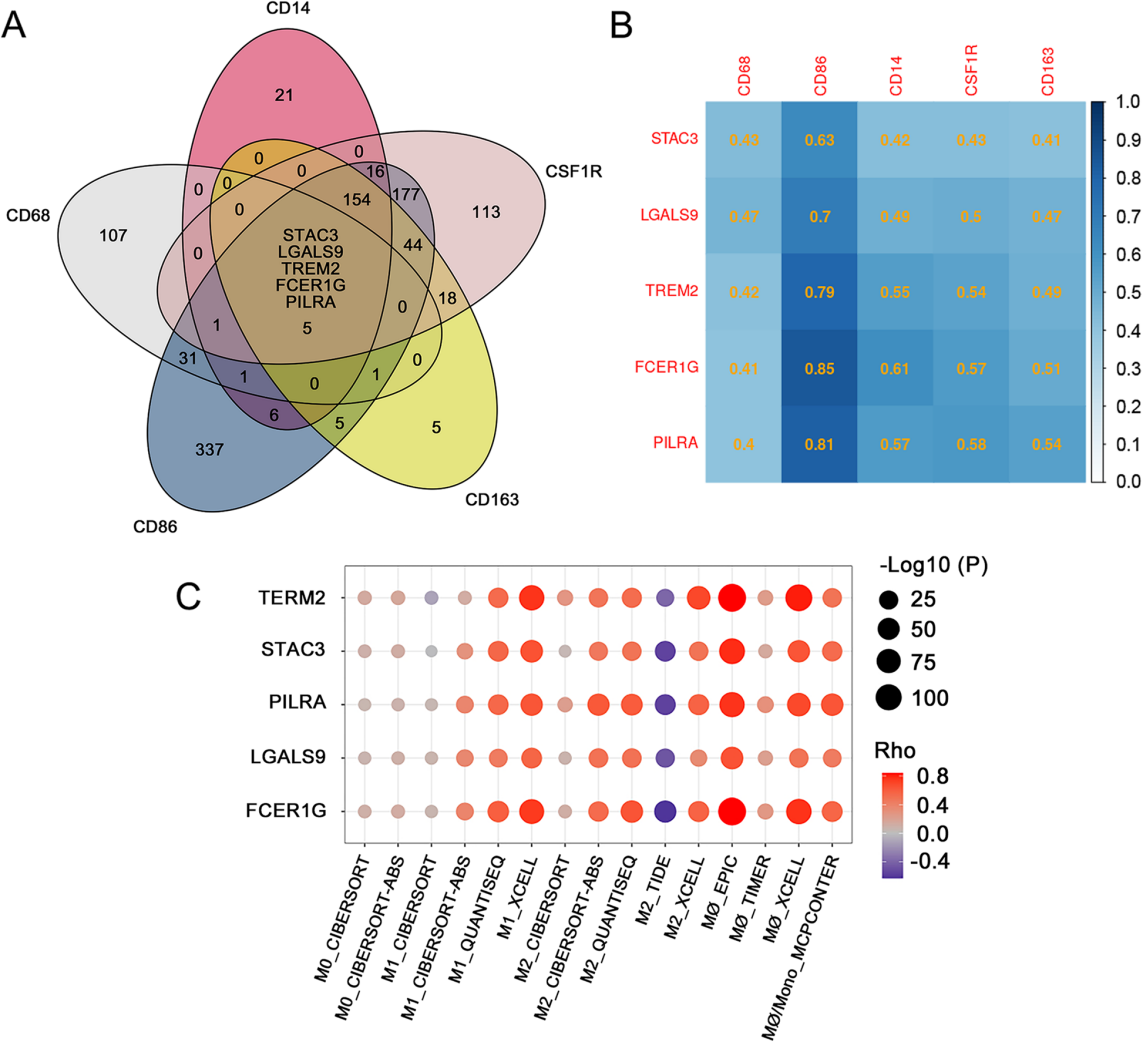
The correlation between DEGs and macrophage markers in TCGA-KIRC database. **A** The Venn diagram shows the intersection of DEGs related to macrophage markers; each ellipse represents a DEG set strongly correlated with a given macrophage marker on the vertex (*r* > 0.4, *P* < 1e-10). **B** The heat map shows the Pearson’s correlation coefficients of five candidate DEGs with macrophage markers. **C** The bubble chart shows the strong correlation of five candidate DEGs with macrophage infiltration in ccRCC estimated using TIMER 2.0. ccRCC, clear cell renal cell carcinoma; DEGs, differentially expressed genes; TCGA-KIRC, The Cancer Genome Atlas-kidney renal clear cell carcinoma

TCGA-KIRC data, including 538 tumors and 72 NATs, were downloaded using the TCGAbiolinks R-package [18]. For data preprocessing, 167 tumor samples were excluded based on the standard of <60% tumor purity using the TCGAtumor_purity function of the package. The remaining data were further normalized using the GC-content method [19] and genes with expression levels <25% of the average levels were excluded. After data preprocessing, the expression data of 13,125 genes from 363 tumor samples and 72 NATs were used for DEGs analysis using the R-package Limma. Using a threshold of the absolute value of log fold change >0.1 and an adjusted P-value <1e-10, it was discovered that 3,323 genes were differentially expressed between tumors and NATs (Fig. 1). Moreover, we selected the five most cited [22–25] macrophage biomarkers (i.e., *CD14*, *CD68*, *CD86*, *CD163*, and colony stimulating factor 1 receptor [*CSF1R*]) and calculated their Pearson’s correlation coefficient with the discovered DEGs. Thus, a set of correlated DEGs was generated for each of the five macrophage biomarkers. For the detection of genes closely related to TAMs, correlated DEGs with a Pearson’s correlation coefficient>0.4 and *P*-value < 1e-10 were considered strongly correlated DEGs (An additional table file shows this in more detail (see Additional file 3)). The Venn diagram intersection analysis revealed that five candidate DEGs were closely related to all selected macrophage biomarkers, namely SH3 and cysteine rich domain 3 (*STAC3*), galectin 9 (*LGALS9*), triggering receptor expressed on myeloid cells 2 (*TREM2*), Fc fragment of IgE receptor Ig (*FCER1G*), and paired immunoglobin like type 2 receptor alpha (*PILRA*) (Fig. 2A, B). For validation of our findings, the web-based tool TIMER 2.0 [26] was employed to calculate the correlation score between the abundance of macrophage infiltrates in ccRCC and the expression levels of each candidate DEG using TCGA-KIRC dataset. For each entered candidate DEG, ≥13 of 15 algorithms showed a positive correlation (*P* < 0.05, *ρ* > 0) (Fig. 2C).

### *FCER1G* was abundantly expressed in ccRCC and contributed to poor prognosis in patients receiving anti-PD-1 treatment

**Fig. 3:**
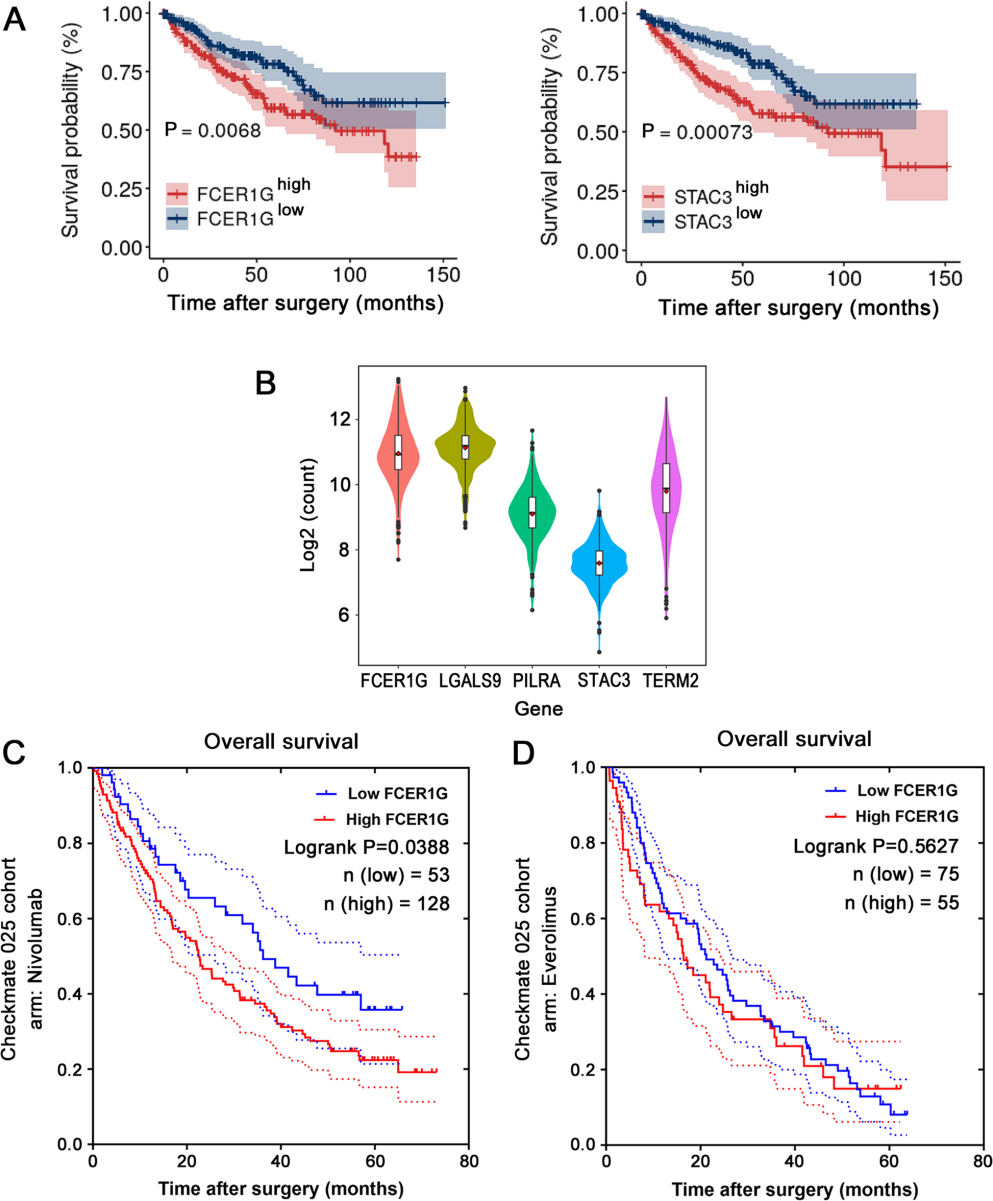
*FCER1G* was abundantly expressed in ccRCC and indicated poor prognosis in patients receiving anti-PD-1 treatment. **A** Kaplan–Meier curves revealing that high expression of *STAC3* and *FCER1G* were related to poor overall survival in patients with ccRCC from TCGA-KIRC. **B** The violin plot shows that *STAC3* has the lowest expression levels among the five candidate DEGs. **C** The Kaplan–Meier survival curve shows that high *FCER1G* expression was related to poor overall survival in patients treated with Nivolumab in the CheckMate 025 clinical trial, but not in **D** those treated with Everolimus in the same research study. ccRCC, clear cell renal cell carcinoma; DEG, differentially expressed gene; FCER1G, Fc fragment of IgE receptor Ig; PD-1, programmed cell death 1; STAC3, SH3 and cysteine rich domain 3; TCGA-KIRC, The Cancer Genome Atlas-kidney renal clear cell carcinoma

To determine their clinical importance, patients with ccRCC from TCGA-KIRC database were divided into two groups according to the median expression levels of each candidate DEG. A Kaplan–Meier survival curve was drawn to examine the difference in OS between the two groups. Among the five identified macrophage-related DEGs, only *STAC3* (*P* = 0.00073) and *FCER1G* (*P* = 0.0068) showed a significant (*P* < 0.05) prognostic difference between the groups (Fig. 3A). Of note, patients with different *STAC3* expression levels exhibited a more significant diversity in OS. However, *STAC3* showed the lowest average expression in ccRCC tissue among all five DEGs (Fig. 3B). Therefore, we selected *FCER1G* as the most pronounced macrophage-related DEG with prognostic value. To further investigate the prognostic value of FCER1G, we downloaded the bulk RNA-seq and clinical data of the CheckMate 025 clinical trial, based on which Nivolumab (anti-PD-1) was approved by the Food and Drug Administration for the treatment of ccRCC [20]. In this dataset, 181 and 130 patients with ccRCC were treated with Nivolumab and Everolimus (MTOR inhibitor), respectively. The best cut-off value of *FCER1G* for each group was derived by the ROC curve (Data not shown). The ROC curve revealed that the best cut-off value for FCER1G expression in Nivolumab arm was 33.34. For Everolimus arm, The ROC curve showed that the best cut-off value for FCER1G expression was 34.27. The Kaplan–Meier survival curve showed that following the administration of Nivolumab, patients with a higher *FCER1G* expression levels were associated with a worse prognosis (Fig. 3C). In contrast, this tendency was not evident in those treated with Everolimus (Fig. 3D).

### *FCER1G* and macrophage markers exerted a synergistic effect on predicting the survival of patients with ccRCC, and high *FCER1G* expression was related to suppression of T lymphocytes

**Fig. 4:**
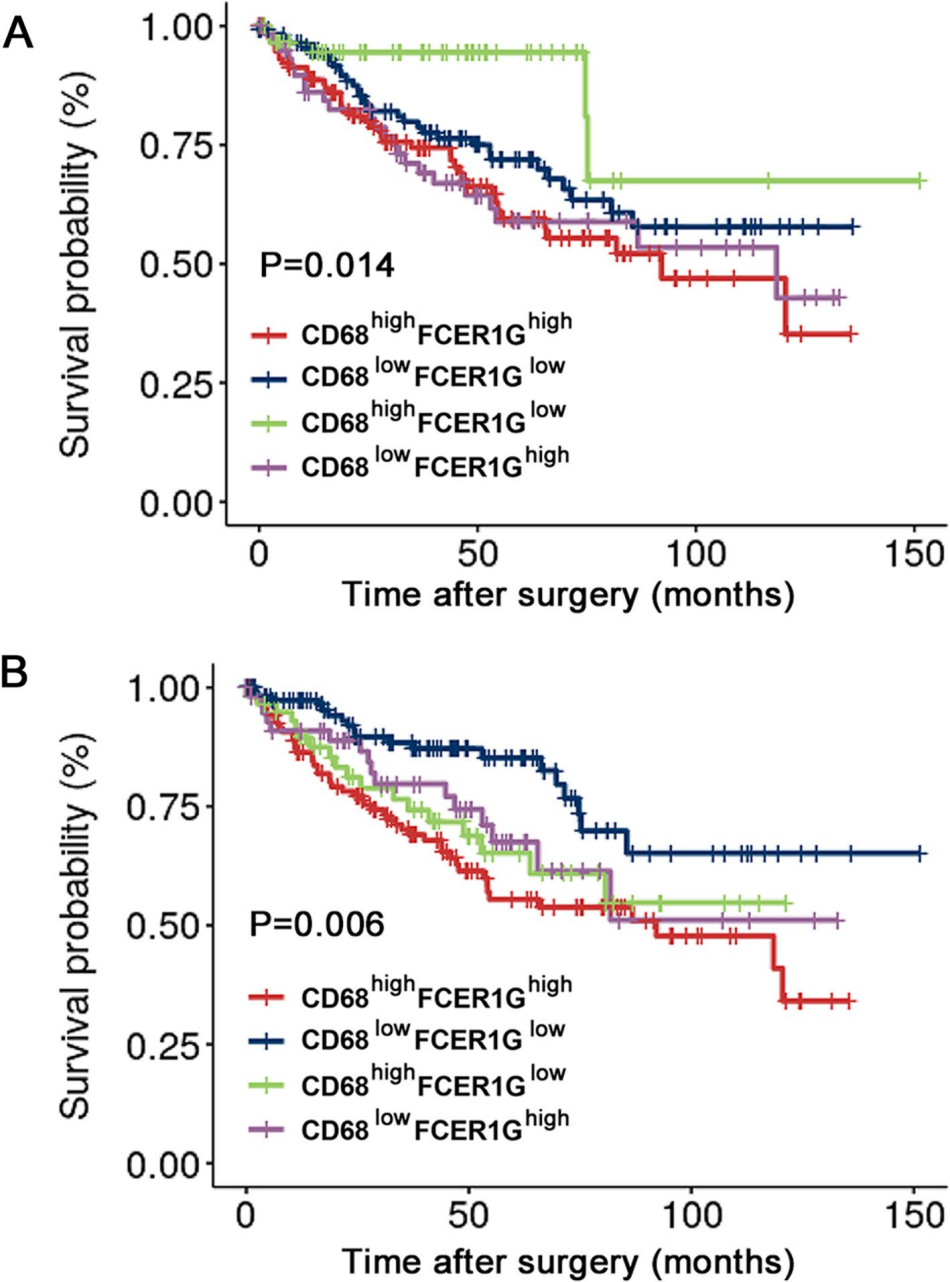
*FCER1G* and macrophage markers exerted a synergistic effect in predicting ccRCC survival. The Kaplan–Meier survival curve shows the combination of *FCER1G* and macrophage marker *CD163* (**A**) or *CD68* (**B**) better predicts patient survival in TCGA-KIRC. ccRCC, clear cell renal cell carcinoma; FCER1G, Fc fragment of IgE receptor Ig; TCGA-KIRC, The Cancer Genome Atlas-kidney renal clear cell carcinoma

**Fig. 5:**
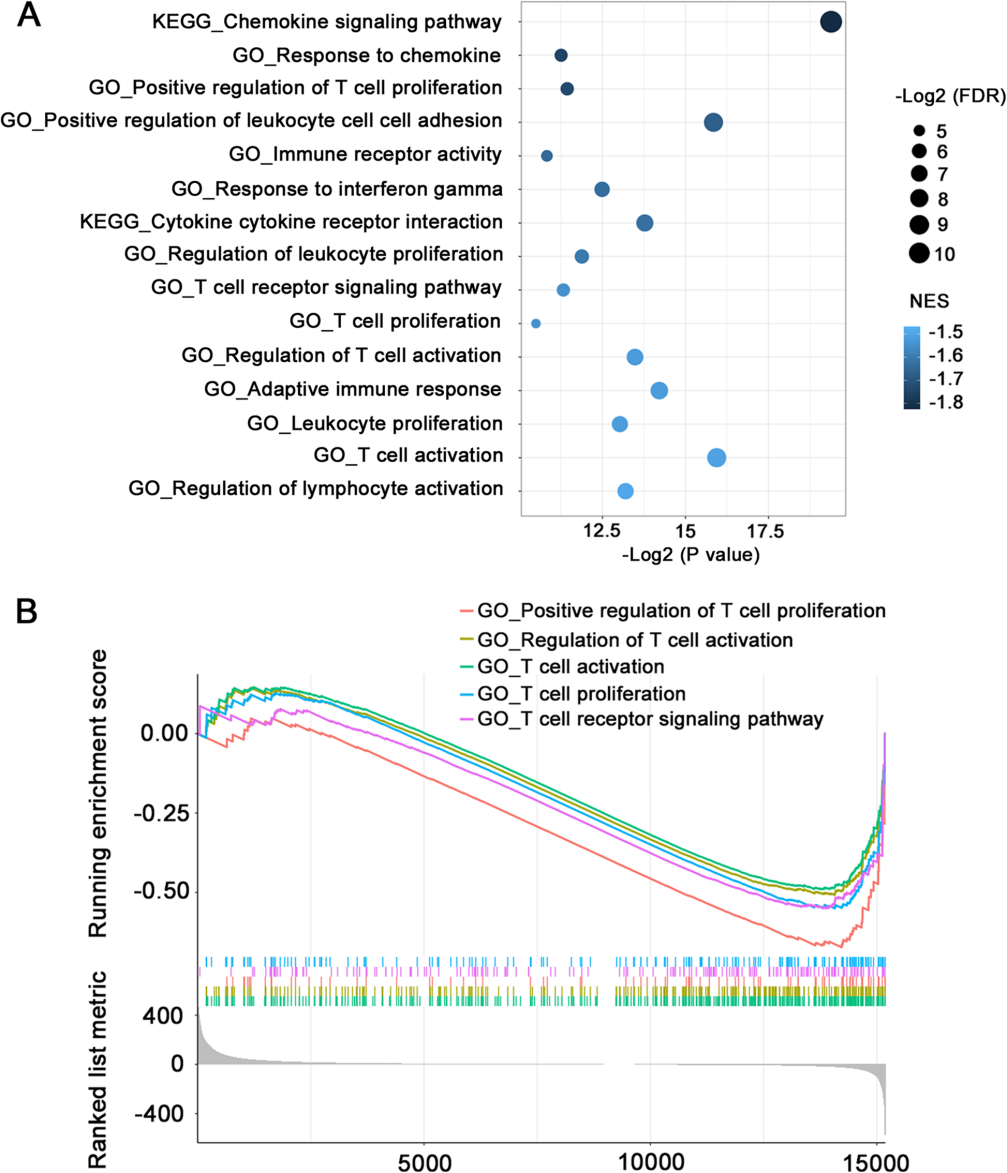
High *FCER1G* expression was related to suppression of T lymphocytes. **A** The bubble chart shows the immune-related gene sets negatively enriched by high *FCER1G* expression using TCGA-KIRC database; the gene sets are arranged in accordance with their normalized enrichment score (NES). **B** The GSEA plot shows the T cell suppression-related gene sets negatively enriched by high *FCER1G* expression and their running enrichment score. FCER1G, Fc fragment of IgE receptor Ig; GSEA, gene set enrichment analysis TCGA-KIRC, The Cancer Genome Atlas-kidney renal clear cell carcinoma

To the best of our knowledge, there are no studies exploring the exact role of *FCER1G* in ccRCC tumor immunity. According to the above-mentioned results, we investigated the potential synergistic effect of *FCER1G* expression and macrophage presence in ccRCC. We employed the median expression levels of *FCER1G* and macrophage markers to classify patients from TCGA-KIRC database into four expression groups: *FCER1G*^high^, *Marker*^high^; *FCER1G*^high^, *Marker*^low^; *FCER1G*^low^, *Marker*^high^ and *FCER1G*^low^, *Marker*^low^. In the groups using *CD68* (Fig. 4A) and *CD163* (Fig. 4B) as classification markers, the combined indicator showed better survival stratification in the Kaplan–Meier survival method. The best stratification was observed with the combination of *CD68* and *FCER1G* (Fig. 4B). Patients with low expression of both *FCER1G* and *CD68* had the longest OS, whereas those with high expression of both *FCER1G* and *CD68* were linked to the worst clinical prognosis. To further investigate the underlying mechanisms of *FCER1G* in ccRCC, we performed GSEA by mapping the gene phenotype plotted according to the *FCER1G* expression levels in TCGA-KIRC with the data obtained from the Gene Ontology and Kyoto Encyclopedia of Genes and Genomes database. Only gene sets with false discovery rate and nominal P-values <0.05 were considered significantly enriched (An additional table file shows this in more detail (see Additional file 5)). Most gene sets negatively enriched by high *FCER1G* expression were related to immune response (Fig. 5A). Notably, gene sets related to T cell function were the most common among all immune response gene sets (Fig. 5B). The results of the GSEA suggested that high *FCER1G* expression in ccRCC is functionally correlated with the suppression of T lymphocytes. This finding is also consistent with the survival curve of patients treated with Nivolumab in the CheckMate 025 trial.

### Prognostic value of FCER1G and CD68 in retrospectively collected ccRCC samples

**Fig. 6:**
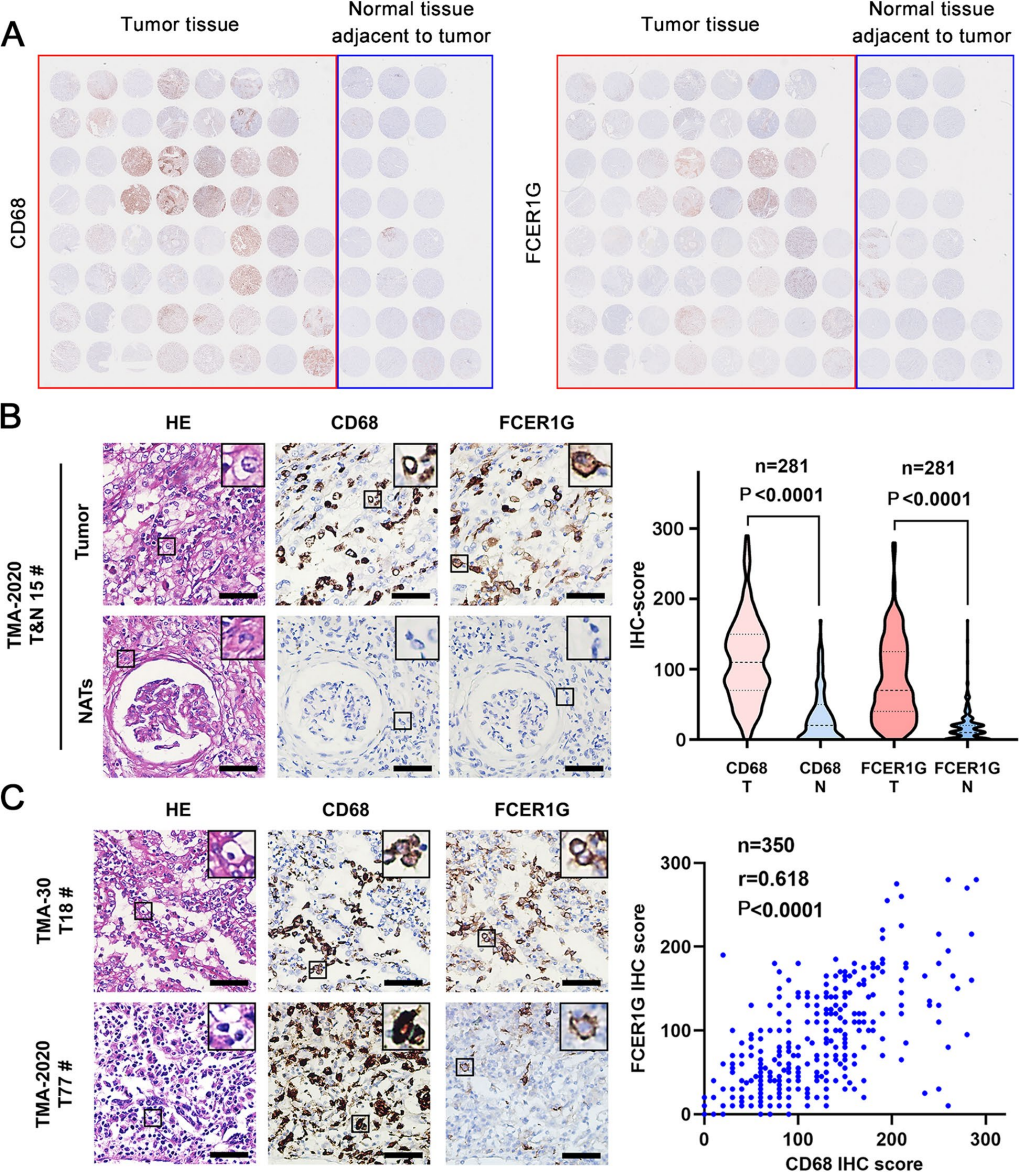
The expression characteristics of FCER1G and CD68 in retrospectively collected ccRCC samples. **A **Thumbnail of TMA-30, showing the expression patterns of CD68 and FCER1G in tumor tissue and normal tissue adjacent to tumor. **B** The representative samples and violin plot reveal the high expression of CD68 and FCER1G in tumor tissue compared with NATs. **C** The representative samples and scatter diagram show the correlation of CD68 and FCER1G in ccRCC samples. ccRCC, clear cell renal cell carcinoma; FCER1G, Fc fragment of IgE receptor Ig; NATs, normal tissues adjacent to tumor

**Fig. 7:**
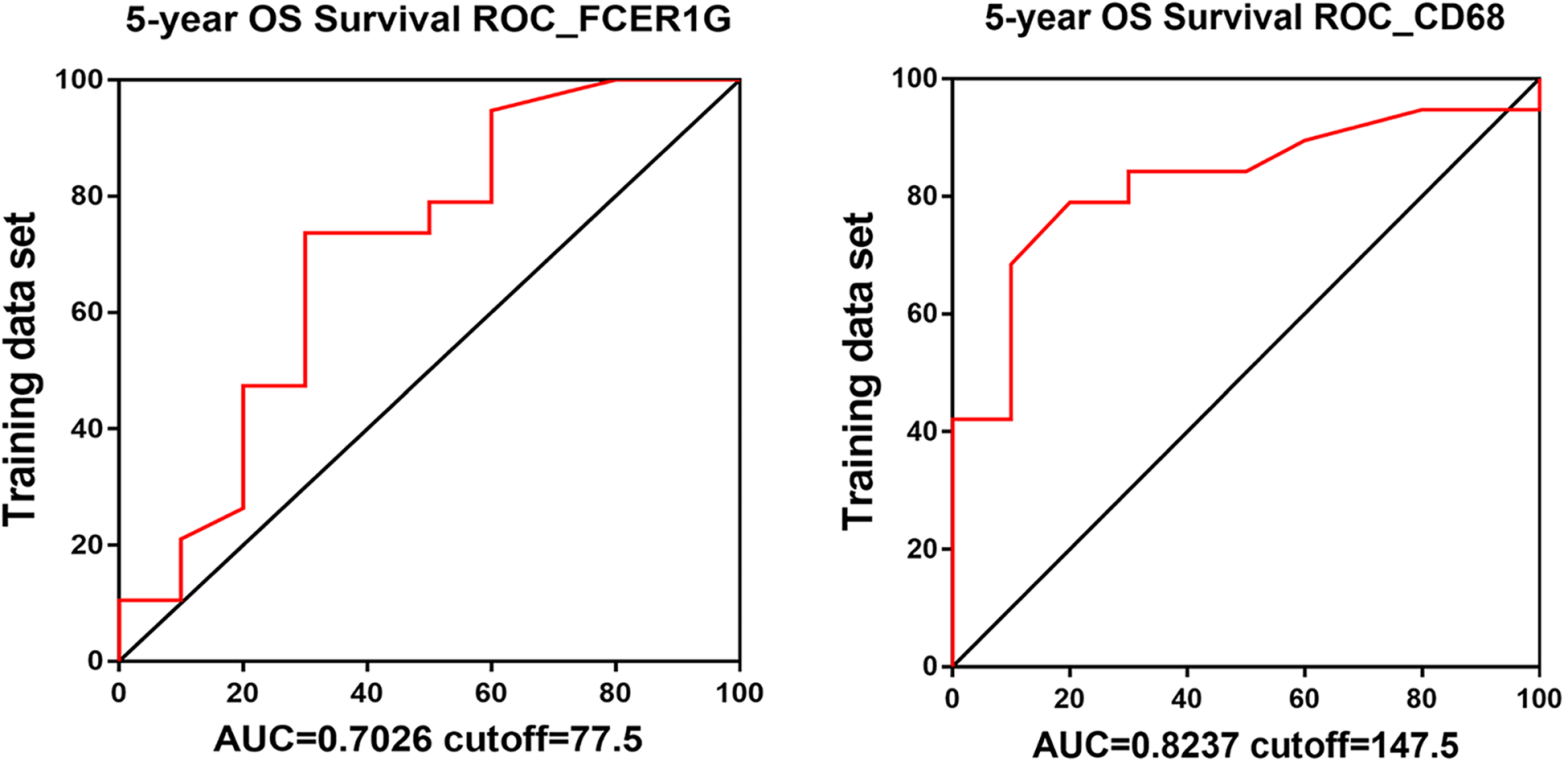
Receiver operating characteristic (ROC) curve based on the CD68 and FCER1G IHC-score. Receiver operating characteristic (ROC) curve for the evaluation of 5-year overall survival based on the CD68 and FCER1G IHC-score of patients with ccRCC from the training set (TMA-30). ccRCC, clear cell renal cell carcinoma; FCER1G, Fc fragment of IgE receptor Ig; IHC, immunohistochemistry; TMA, tissue microarray

**Fig. 8:**
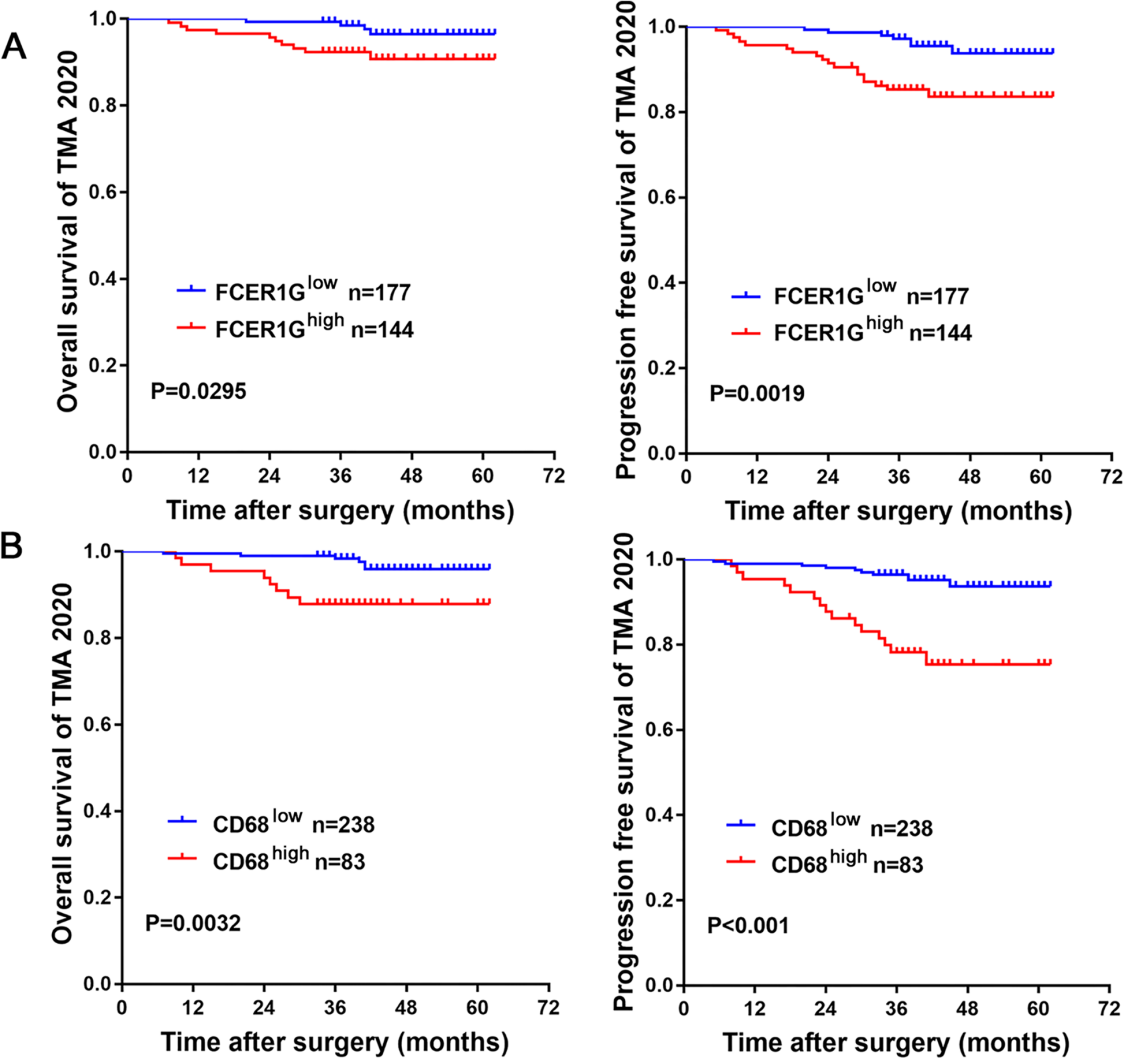
Prognostic value of FCER1G and CD68 in retrospectively collected ccRCC samples. **A** The Kaplan–Meier survival curve shows that high expression of FCER1G was related to poor overall survival and progression-free survival in patients from the validation set (TMA-2020). **B** The Kaplan–Meier survival curve shows that high expression of CD68 was related to poor overall survival and progression-free survival in patients from the validation set (TMA-2020). ccRCC, clear cell renal cell carcinoma; FCER1G, Fc fragment of IgE receptor Ig; IHC, immunohistochemistry; TMA, tissue microarray

To further validate our findings in clinical setting, we collected samples from 350 patients with ccRCC (Table 1) (Additional table files show this in more detail (see Additional file 1 and 2)) who underwent either open or laparoscopic surgery from 2010 to 2018 in Shanghai Changhai Hospital. Samples stained with hematoxylin-eosin were examined, and nine pieces of TMA were produced (TMA-30 and TMA-2020 NO.1-8, described in Methods). IHC staining was performed to determine the expression of FCER1G and CD68 in ccRCC using successive sections (thickness: 4 μm) (Fig. 6A). Next, the IHC-score (described in Methods) of each sample was evaluated. Higher expression of both FCER1G and CD68 was detected in tumors compared with NATs (Fig. 6B). Meanwhile, the expression levels of FCER1G and CD68 were strongly correlated in ccRCC tissues (Fig. 6C), with a Pearson’s correlation coefficient of 0.618. Although the correlation of expression was strong, we observed that the expression pattern of FCER1G and CD68 differed in a considerable portion of samples (Fig. 6A, C lower left); hence, the types of cells that FCER1G and CD68 represent varied. Owing to the long duration of the follow-up and completeness of OS and PFS, TMA-30 was used as the training group to determine the best cut-off value for the FCER1G and CD68 IHC-score. Using 5-year OS as primary outcome, the ROC curve showed that the best IHC-score cut-off value for FCER1G was 77.5, with an AUC of 0.7026. For CD68, the best IHC-score cut-off value was 147.5, with an AUC of 0.8237 (Fig. 7) (Tables 2, 3). Subsequently, a Kaplan–Meier survival curve was drawn for TMA-2020 NO.1-8 to evaluate the prognostic value of FCER1G or CD68. Patients with high expression levels of FCER1G (Fig. 8A) or CD68 (Fig. 8B) were associated with inferior OS and PFS. Univariate and multivariate Cox regression analyses were performed to further determine whether FCER1G and CD68 were independent risk factors for evaluating OS and PFS in patients with ccRCC. Both FCER1G and CD68 were associated with shorter OS and PFS in Univariate Cox regression analysis. Following multivariable adjustment (i.e., age, sex, Fuhrman grade, TNM stage, and T category), CD68 was identified as an independent risk factor for the PFS of patients with ccRCC (Tables 4, 5).

**Table 2:** Correlation between FCER1G expression and clinical characteristics of patients with ccRCC in TMA-2020 NO.1-8 (*n* = 321)

Characteristics	FCER1G expression in TMA-2020 NO.1-8		Total (*n* = 321)	*P*-value
	High expression (*n* = 177)	Low expression (*n* = 144)		
**Age**				0.8798
<60 years	103	85	188	
≥60 years	74	59	133	
**Sex**				0.0663
Male	116	108	224	
Female	61	36	97	
**Fuhrman grade**				**<0.0001**
1–2	158	103	261	
3–4	19	41	60	
**TNM stage**				**0.0046**
I–II	161	114	275	
III–IV	15	30	45	
NA	1	0	1	
**T category**				**0.0008**
T1a–T1b	152	100	252	
T2a–T4	24	44	68	
NA	1	0	1	
**Overall survival**				**0.0291**
−	141	108	249	
+	5	12	17	
NA	31	24	55	
**Progression-free survival**				**0.0010**
−	138	98	236	
+	8	22	30	
NA	31	24	55	

*ccRCC* clear cell renal cell carcinoma; *FCER1G* Fc fragment of IgE receptor Ig; *NA* not applicable; *TMA* tissue microarray

**Table 3:** Correlation between CD68 expression and clinical characteristics of patients with ccRCC in TMA-2020 NO.1-8 (*n* = 321)

Characteristics	CD68 expression in TMA-2020 NO.1-8		Total (n = 321)	*P*-value
	High expression (*n* = 238)	Low expression (*n* = 83)		
**Age**				0.4993
<60 years	142	46	188	
≥60 years	96	37	133	
**Sex**				0.0914
Male	160	64	224	
Female	78	19	97	
**Fuhrman grade**				**<0.0001**
1–2	207	54	261	
3–4	31	29	60	
**TNM stage**				**0.0024**
I–II	213	62	275	
III–IV	24	21	45	
NA	1	0	1	
**T category**				**0.0117**
T1a–T1b	196	56	252	
T2a–T4	41	27	68	
NA	1	0	1	
**Overall survival**				**0.0282**
−	191	58	249	
+	9	8	17	
NA	38	17	55	
**Progression-free survival**				**<0.0001**
−	186	49		
+	14	16		
NA	38	17	55	

*ccRCC* clear cell renal cell carcinoma; *NA* not applicable; *TMA* tissue microarray

**Table 4:** Univariate and multivariate Cox regression analyses of patient characteristics with overall survival

Characteristics	Univariate		Multivariate	
	HR (95% CI)	*P*-value	HR (95% CI)	*P*-value
**Age (<60 vs. ≥60 years)**	2.1 (0.72–6.00)	0.18		
**Sex (female vs. male)**	1.6 (0.44–5.70)	0.48		
**Fuhrman grade (1–2 vs. 3–4)**	4.5 (1.6–13.0)	0.005	4.027 (1.3926–11.6430)	0.0101
**TNM stage (1–2 vs. 3–4)**	4.8 (1.6–14.0)	0.0049	3.092 (0.9729–9.8270)	0.0557
**T category (T1a–1b vs. T2a–T4)**	6.1 (2.9–13.0)	1.5E-06	5.0043 (1.8022–13.8960)	0.002
**CD68 (low vs. high)**	4.3 (1.5–12.0)	0.0069	2.5303 (0.7912–8.0920)	0.11758
**FCER1G (low vs. high)**	3.4 (1.1–11.0)	0.04	1.5414 (0.4111–5.7790)	0.52102

*CI* confidence interval; *FCER1G* Fc fragment of IgE receptor Ig; *HR* hazard ratio

**Table 5:** Univariate and multivariate Cox regression analyses of patient characteristics with progression-free survival

Characteristics	Univariate		Multivariate	
	HR (95% CI)	*P*-value	HR (95% CI)	*P*-value
**Age (<60 vs. ≥60 years)**	1.5 (0.69–3.10)	0.32		
**Sex (female vs. male)**	1.5 (0.6–3.7)	0.38		
**Fuhrman grade (1–2 vs. 3–4)**	3.9 (1.8–8.2)	0.0005	3.621 (1.6718–7.8450)	0.0011
**TNM stage (1–2 vs. 3–4)**	6.6 (3–14)	1.7E-06	4.886 (2.1503–11.1010)	0.00015
**T category (T1a–1b vs. T2a–T4)**	5.5 (3.3–9.1)	1E-10	3.7106 (1.6951–8.1230)	0.00104
**CD68 (low vs. high)**	4.9 (2.3–11.0)	0.000047	2.9592 (1.2744–6.8710)	0.0116
**FCER1G (low vs. high)**	4 (1.7–9.5)	0.0016	1.7784 (0.6478–4.8820)	0.26382

*CI* confidence interval; *FCER1G* Fc fragment of IgE receptor Ig; *HR*, hazard ratio

### Combination of CD68 and FCER1G expression resulted in a better prognostic stratification in patients with ccRCC

**Fig. 9:**
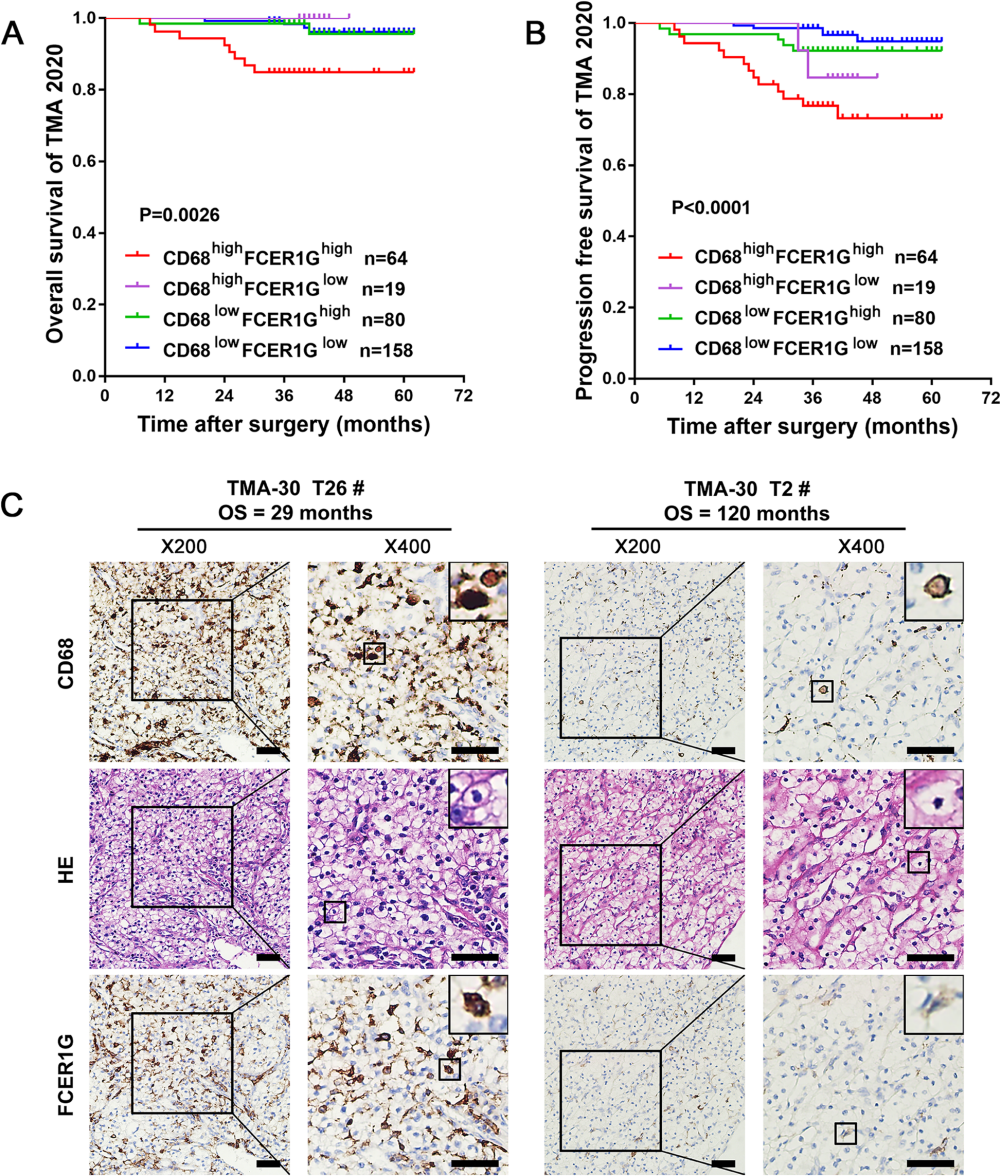
Combining CD68 and FCER1G expression resulted in better prognostic stratification in patients with ccRCC. The Kaplan–Meier survival curve reveals that combining CD68 and FCER1G expression more accurately determines the overall survival (**A**) and progression-free survival (**B**) in patients with ccRCC from the validation set (TMA-2020). **C** Immunohistochemistry results of representative samples show an inferior overall survival in patients with high expression of both CD68 and FCER1G. ccRCC, clear cell renal cell carcinoma; FCER1G, Fc fragment of IgE receptor Ig

**Fig. 10:**
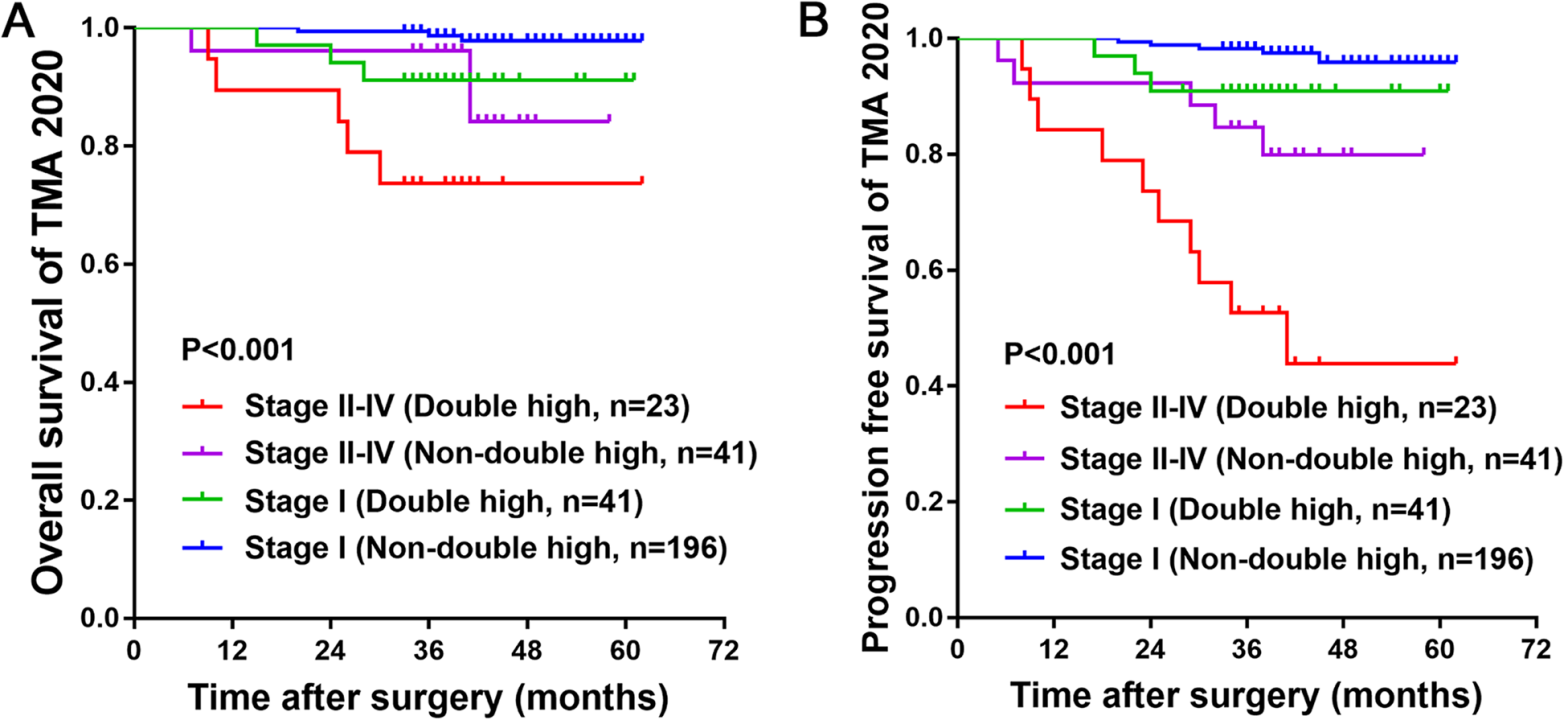
Integration of CD68 and FCER1G expression into TNM staging exhibited high accuracy in assessing prognoses. The Kaplan–Meier survival curve shows that patients with high expression of both CD68 and FCER1G (double high) were associated with worse overall survival (**A**) and progression-free survival (**B**) compared with the remaining patients (non-double high) from the same TNM stage group. ccRCC, clear cell renal cell carcinoma; FCER1G, Fc fragment of IgE receptor Ig

**Fig. 11:**
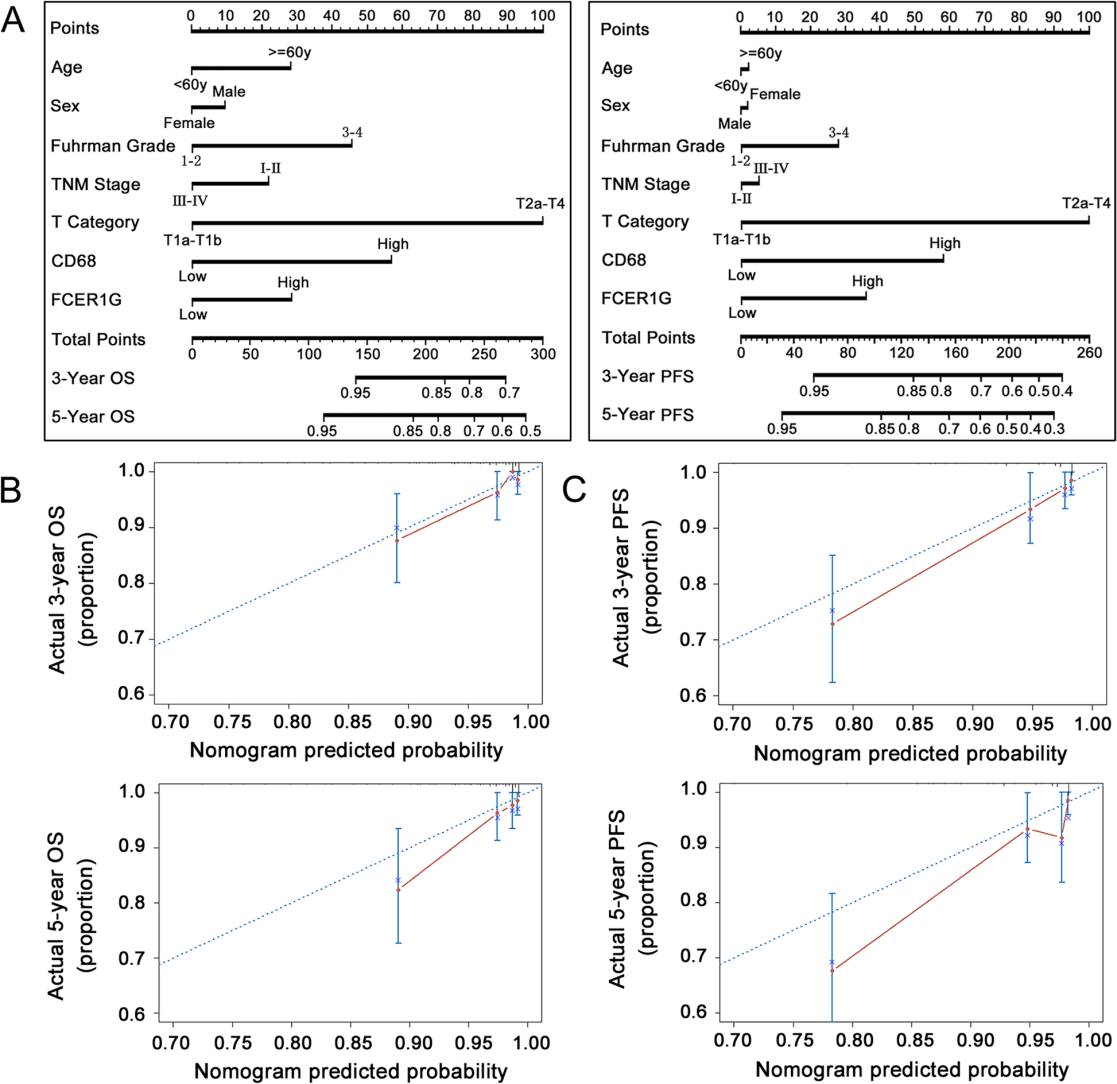
Nomograms for predicting OS and PFS of patients with ccRCC. **A **Nomograms for predicting OS and PFS of patients with ccRCC at 3 or 5 years after surgery. Calibration plots of the nomogram for the prediction of 3-year and 5-year OS (**B**) and PFS (**C**). ccRCC, clear cell renal cell carcinoma; OS, overall survival; PFS, progression-free survival

To further test the possible synergistic effect of FCER1G and CD68 expression in predicting the prognosis of ccRCC, 321 patients in TMA-2020 were classified into four groups using the cut-off values determined from the ROC curve: FCER1G^high^, CD68^high^; FCER1G^high^, CD68^low^; FCER1G^low^, CD68^high^ and FCER1G^low^, CD68^low^. The Kaplan–Meier survival curve showed a significant difference in survival between different groups in terms of OS (Fig. 9A) and PFS (Fig. 9B). The group with high expression of both FCER1G and CD68 showed the worst prognosis (Fig. 9C). Furthermore, we examined the prognostic accuracy of FCER1G and CD68 expression versus that of established indicators in patients with ccRCC. Concordance index analysis was used in the validation cohort (*n* = 321), which demonstrated that the integration of CD68 and FCER1G expression into the established prognostic indicators exhibited a higher concordance index value than any of these indicators alone (Table 6). In the same TNM stage group, patients with high expression of both CD68 and FCER1G (double high) showed worse OS (Fig. 10A) and PFS (Fig. 10B) compared with the remaining patients (non-double high). This may assist physicians in distinguish high-risk patients following surgery for ccRCC. Finally, we constructed nomograms to predict OS and PFS in patients with ccRCC at 3 and 5 years (Fig. 11A). Calibration plots of the nomograms for the prediction of 3- and 5-year OS (Fig. 11B) and PFS (Fig. 11C) are presented below.

**Table 6:** Concordance index analysis

Characteristics	Overall survival	Progression-free survival
	Validation cohort (*n* = 321)	Validation cohort (*n* = 321)
**Fuhrman grade (1–2 vs. 3–4)**	0.4970 (0.4055–0.5884)	0.4893 (0.3917–0.5869)
**TNM stage (1–2 vs. 3–4)**	0.5265 (0.4153–0.6377)	0.5217 (0.3989–0.6444)
**T category (T1a–1b vs T2a–T4)**	0.5419 (0.4502–0.6335)	0.5438 (0.4439–0.6437)
**CD68 (low vs. high)**	0.5616 (0.4802–0.6431)	0.5420 (0.4546–0.6295)
**CD68 + Fuhrman grade**	0.5236 (0.4557–0.5917)	0.5194 (0.4480–0.5908)
**CD68 + TNM stage**	0.5450 (0.4743–0.6157)	0.5404 (0.4653–0.6156)
**CD68 + T category**	0.5462 (0.4795–0.6128)	0.5451 (0.4748–0.6155)
**FCER1G (low vs. high)**	0.5959 (0.5221–0.6696)	0.5906 (0.5141–0.6669)
**FCER1G + Fuhrman grade**	0.5537 (0.4922–0.6153)	0.5653 (0.4992–0.6314)
**FCER1G + TNM stage**	0.5693 (0.5050–0.6335)	0.5676 (0.5005–0.6348)
**FCER1G + T category**	0.5686 (0.5084–0.6288)	0.5688 (0.5059–0.6318)
**CD68 + FCER1G**	0.5753 (0.5145–0.6361)	0.5668 (0.5032–0.6305)

*FCER1G*, Fc fragment of *IgE* receptor Ig

Discussion In this study, we reported five DEGs in ccRCC that are strongly correlated with the expression of macrophage markers (i.e., *LGALS9*, *PILRA*, *TREM2*, *STAC3*, and *FCER1G*). *LGALS9* encodes galectin-9 which is a beta-galactoside-binding protein participating in cell-cell and cell-matrix interactions [27]. *PILRA* encodes PILRα, which is an immune inhibitory receptor possessing an immunoreceptor tyrosine-based inhibitory motif (ITIM) in its cytoplasmic domain [28]. *TERM2*, the coding gene for triggering a receptor expressed on monocytes 2, is responsible for the uptake of β-amyloid oligomers by microglia [29]. These three genes are highly involved in immune-related pathways. However, it has been reported that *STAC3* encodes an important molecule involved in excitation–contraction coupling that regulates the contraction of skeletal muscles [30]. Although *STAC3* showed prognostic importance in TCGA-KIRC database, its underlying mechanism warrants further investigation.

*FCER1G* encodes the Fc receptor γ chain, which was firstly reported in 1990 to be the third subunit of the high-affinity immunoglobulin E (IgE) receptor (Fcε RI) [31]. Later, it was demonstrated that the Fc receptor γ chain is a common component of Fc receptors widely expressed in different types of immune cells and participates in a variety of immune responses, such as phagocytosis and cytokine release [32]. Under physiological conditions, Fc receptors eliminate pathogens and antigens by binding to the crystallizable fragment of immunoglobulins. In contrast, under pathological conditions, they can lead to abnormal immune responses such as IgE-dependent allergy [33].

It has been reported that, in the viral infection process, FCER1G-expressing natural killer cells could respond to the over activation of CD8^+^ T cells and suppress their function [34]. A reduction in regulatory T cells was also observed in *FCER1G* -deficient mice [35]. Similarly, GSEA analysis revealed that high *FCER1G* expression is related to suppression of T cell activation and proliferation. In the CheckMate 025 trial, high *FCER1G* expression was suggestive of poor prognosis in patients treated with an anti-PD-1 agent which is a monoclonal antibody that acts by blocking inhibitory transmembrane protein expressed on T cells to in turn stimulate the anti-tumor immune response. However, this association was not observed in patients treated with an MTOR inhibitor that mainly targets the tumor itself. Thus, we suggest that *FCER1G* expression is related to T cell function in ccRCC. However, the specific regulatory mechanism of this effect needs to be further studied.

If the two genes are highly correlated, there is a possibility that the two genes are expressed in the same type of cell. The expression of the Fc receptor γ chain in macrophages has been previously reported by others [36, 37]. However, by IHC staining of the successive sections from our tissue microarray, we showed that the spatial orientation of FCER1G and CD68 differed in ccRCC, suggesting that FCER1G is located on macrophages and other types of cells. The Kaplan–Meier survival analysis also showed the worst outcome in the group with high mRNA and protein expression of both *FCER1G* and *CD68*, suggesting disparity in their function in *FCER1G*- and *CD68*-expressing cells. Therefore, it is important to examine the underlying molecular mechanism that governs the synergistic effect of *FCER1G* and *CD68* in ccRCC. The Fc receptor γ chain is an important component of allergy-related Fcε RI, which is abundantly expressed on mast cells and basophils. It is established that the activation of Fcε RI on mast cells and basophils leads to secretion of interleukin 4 (IL4) [38], which is an inducer of a tumor-suppressive macrophage subtype (M2 macrophage) [39]. In this rationale, the high expression of *FCER1G* in ccRCC could be a functional basis for the induction of M2 macrophages in ccRCC by the increased secretion of IL4. Furthermore, M2 macrophages derive their tumor-suppressive function partly by suppressing cytotoxic T cells. This may explain the correlation of *FCER1G* with both macrophages and T cell function.

The prognostic role of *FCER1G* expression in ccRCC has been previously discovered through protein–protein interaction networks [40] or weighted gene co-expression network [41] analysis of DEGs using open-source bulk RNA-sequencing data of ccRCC. Another study used the pre-established ESTMATE algorithm [42] to assess the genes with prognostic value in the immune microenvironment of ccRCC, identifying *FCER1G* as a candidate gene [43]. Nevertheless, there are no studies conducted so far that investigated the correlation of *FECER1G* expression with macrophages or validated theirfindings in a large clinical cohort. Although the GSEA analysis of high *FECER1G* expression in TCGA-KIRC was performed by Wang and his colleague, they failed to fully interpret the function of *FCER1G* in suppressing T cells in ccRCC [40]. Unlike previously studies, we identified *FCER1G* as prognosis marker of ccRCC based on its correlation with macrophage markers.

This study was characterized by certain limitations. Firstly, the relationship of *FCER1G* expression with macrophage infiltration and suppressed T cell function are simply concluded by the correlation analysis. Further experiments are required to elucidate the exact cell type in which *FCER1G* is overexpressed. This is urgently needed, as it may provide insight into the underlying mechanism of *FCER1G* expression interaction with tumor immunity and affect the prognosis of ccRCC. Secondly, the patient samples included in the study were collected in a single clinical center mainly from 2016 to 2018; most of those patients in TMA-2020 did not reach a 5-year follow-up (range: 33–62 months, median follow-up: 42 months). Moreover, the death rate in TMA-2020 was only 5.30%, impacting the statistical evaluation of several analyses, such as the multivariate Cox regression. We also observed high *FCER1G* and *CD68* expression levels in patients lost to follow-up, indicating that the death rate in the high *FCER1G* and *CD68* expression group may be underestimated. Multi-centered studies involving large cohorts of patients with ccRCC and a long term follow-up may lead to more accurate conclusions.

## Conclusions

In this study, we used TCGA-KIRC database to investigate genes correlated with macrophages markers and elucidate the association between *FCER1G* expression and tumor-infiltrating macrophages. *FCER1G* was abundantly expressed in tumor tissues and showed prognostic importance in patients with ccRCC treated with nivolumab, but not in those treated with everolimus. Combination of *FCER1G* and macrophage biomarker *CD68* can result in better prognostic stratification of patients with ccRCC from TCGA-KIRC. Furthermore, we performed GSEA and predicted the T cell-suppressive role of *FCER1G* in ccRCC. We also validated that FCER1G and CD68 are both highly expressed in tumor tissue and correlated with each other. Higher expression of CD68 or FCER1G in ccRCC tissue indicated shorter OS and PFS, and patients with high expression of both CD68 and FCER1G had the worst outcome. Integration of FCER1G and CD68 expression into TNM staging exhibited high accuracy in assessing patients with different prognoses following surgery for ccRCC.

## Supplementary Information

**Additional file 1.** Detail information of TMA-30.

**Additional file 2.** Detail information of TMA-2020 NO.1-8.

**Additional file 3.** Differentially expressed genes correlated with macrophage markers.

**Additional file 4.** Gene sets enriched in TCGA-KIRC with high FCER1G expression.

## Data Availability

The datasets used or analyzed during the current study are available from the corresponding author on reasonable request. The RNA sequencing count data of TCGA-KIRC were downloaded using the R package ‘TCGAbiolinks’. Bulk RNA sequencing data of the CheckMate 025 clinical trial are available in the European Genome-Phenome Archive (https://ega-archive.org/): EGAS00001004290, EGAS00001004291, EGAS00001004292.

## Abbreviations

TAMs: Tumor-associated macrophages
ccRCC: clear cell renal cell carcinoma
RCC: renal cell carcinoma
TKIs: tyrosine kinase inhibitors
ICI: immune checkpoint inhibitor
RNA-seq: RNA sequencing
DEGs: differentially expressed genes
TMA: tissue microarray
NATs: normal tissues adjacent to tumor
TCGA-KIRC: The Cancer Genome Atlas-kidney renal clear cell carcinoma
GSEA: gene set enrichment analysis
